# Inefficient Quality Control of Thermosensitive Proteins on the Plasma Membrane

**DOI:** 10.1371/journal.pone.0005038

**Published:** 2009-04-01

**Authors:** Michael J. Lewis, Hugh R. B. Pelham

**Affiliations:** MRC Laboratory of Molecular Biology, Cambridge, United Kingdom; University of Geveva, Switzerland

## Abstract

**Background:**

Misfolded proteins are generally recognised by cellular quality control machinery, which typically results in their ubiquitination and degradation. For soluble cytoplasmic proteins, degradation is mediated by the proteasome. Membrane proteins that fail to fold correctly are subject to ER associated degradation (ERAD), which involves their extraction from the membrane and subsequent proteasome-dependent destruction. Proteins with abnormal transmembrane domains can also be recognised in the Golgi or endosomal system and targeted for destruction in the vacuole/lysosome. It is much less clear what happens to membrane proteins that reach their destination, such as the cell surface, and then suffer damage.

**Methodology/Principal Findings:**

We have tested the ability of yeast cells to degrade membrane proteins to which temperature-sensitive cytoplasmic alleles of the Ura3 protein or of phage lambda repressor have been fused. In soluble form, these proteins are rapidly degraded upon temperature shift, in part due to the action of the Doa10 and San1 ubiquitin ligases and the proteasome. When tethered to the ER protein Use1, they are also degraded. However, when tethered to a plasma membrane protein such as Sso1 they escape degradation, either in the vacuole or by the proteasome.

**Conclusions/Significance:**

Membrane proteins with a misfolded cytoplasmic domain appear not to be efficiently recognised and degraded once they have escaped the ER, even though their defective domains are exposed to the cytoplasm and potentially to cytoplasmic quality controls. Membrane tethering may provide a way to reduce degradation of unstable proteins.

## Introduction

Degradation of misfolded proteins is a key mechanism for cellular maintenance and a major use of the ubiquitin modification system. Misfolded cytoplasmic proteins undergo polyubiquitination with K48-linked chains; these are then recognised by the proteasome and the ubiquitinated proteins degraded [1]–[3]. Newly-synthesised membrane and secretory proteins that fail to fold are recognised in the endoplasmic reticulum, ejected into the cytoplasm and ubiquitinated, before similarly undergoing proteasomal degradation in a process termed ER-associated degradation (ERAD) [4]–[6]. Membrane proteins that reach the Golgi or plasma membrane can also be degraded when necessary; they undergo subsequent addition of single ubiquitins, or K63-linked chains, which direct them into multivesicular bodies for degradation in the lysosome or vacuole [7]–[10].

The key common requirement is that misfolded proteins, in various locations, are recognised as such by an appropriate ubiquitin ligase. In yeast, several have been identified. For ER-associated degradation the membrane-associated RING domain E3 ligases Hrd1 and Doa10 are the prime candidates [11]–[13]. Surprisingly, Doa10 is also implicated in the degradation of some soluble proteins [11], [14], [15]. The nuclear RING domain protein San1 is involved in the destruction of several temperature-sensitive nuclear proteins [16]. Membrane proteins that escape the ER can be recognised by the RING protein Tul1 [17], or by the adaptor Bsd2, which recruits the HECT domain ligase Rsp5 [18]. Both these proteins recognise primarily polar transmembrane regions, as would be exposed by misfolding of the membrane-spanning portion of a polytopic protein, and they serve to target proteins to the vacuole. Both appear to function in the Golgi and endosomes.

Despite these findings, there remains considerable uncertainty over the systems responsible for degradation of abnormal proteins, partly because of redundancy. For example, soluble proteins may aggregate and undergo autophagy as well as being degraded by the proteasome [19], and there may well be multiple enzymes capable of ubiquitinating any given protein.

We have sought to understand the full range of quality control of membrane proteins. Though most mistakes occur during the initial folding process, and thus can be detected at the level of the ER or Golgi, there is at least the potential for damage to proteins after they arrive at their destination. Are plasma membrane proteins constantly monitored for damage, and if so, how are they disposed of? If, for example, the cytoplasmic domain of a plasma membrane protein is damaged, will it be recognised by the cytoplasmic proteasomal system, or targeted to the vacuole? To address this, we have fused temperature sensitive soluble proteins to membrane anchors and investigated their degradation after a temperature shift. Surprisingly, though these proteins are degraded when soluble or attached to the ER, once delivered to the cell surface or vacuolar membrane they are much more stable. It appears that such proteins are quite well tolerated by cells, and there is no quality control system designed to destroy them rapidly.

## Results

### Degradation of cytoplasmic Ura3 fusion proteins

**Figure 1:**
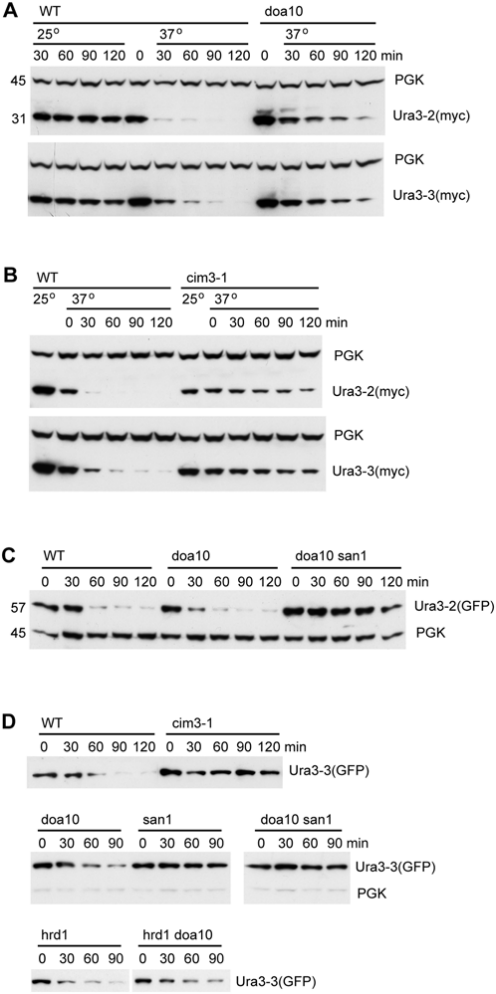
Degradation of temperature-sensitive Ura3 alleles. A. Cells expressing myc-tagged Ura3 proteins were treated with cycloheximide and incubated at 25 or 37°C for the times indicated. Total protein samples were immunoblotted, with phosphoglycerate kinase (PGK) serving as an internal control. Molecular weights (kDa) of the proteins are indicated on the left of the blot B. As A, but with wild-type and *cim3-1* (ts proteasome mutant) cells. Note that for this test cells were incubated at high temperature for one hour before cycloheximide addition (at time 0). C. GFP-tagged Ura3-2 in wild-type and the indicated mutant cells. D. GFP-tagged Ura3-3 in the indicated stains.

Our strategy was first to identify soluble proteins whose metabolic stability could be controlled by a temperature shift, then attach these to a membrane anchor. Initially, we mutagenised a myc-tagged version of Ura3 and isolated temperature-sensitive mutants by their ability to grow at 37°C on 5-fluoro-orotic acid, which kills cells with active Ura3. These mutants were then screened for Ura3 degradation during a cycloheximide chase. Two mutants were obtained with non-conservative changes in distinct regions of the protein: Ura3-2 had Pro144 changed to Leu, and Ura3-3 had Asp243 changed to Gly, as well as the conservative mutation of Lys176 to Arg. The structural consequences of the two mutations are expected to be distinct [20]. Asp243 is at the start of a C-terminal helix of the Ura3 monomer, and its conversion to Gly might destabilise this helix. Pro144 on the other hand is in a turn on the opposite side of the molecule. Figure 1A shows that both these proteins persisted during a cycloheximide chase at 25°C, but after shifting to 37° they were rapidly degraded. In both cases this degradation was dramatically slowed in the temperature sensitive proteasome mutant *cim3-1* (Figure 1B), suggesting that they were subject to proteasomal degradation, presumably ubiquitin-mediated.

Ura3 variants with random peptides appended to them have been shown to be substrates for the Doa10 ubiquitin ligase [21]. Our ts alleles had a longer lifetime in a *doa10* deletion mutant, but the fact that degradation could still be observed implies that Doa10 is not the only ligase contributing to their fate (Figure 1A).

To test whether the metabolic instability of the Ura3 mutants could be extended to a fusion protein, we tagged them with GFP. The GFP fusions were degraded in a temperature-sensitive manner, and this was blocked by the ts proteasome mutant *cim3-1* (shown for Ura3-3(GFP) in Figure 1D), although not significantly in a *doa10* mutant (Figure 1C, D). However, we found that their degradation was substantially reduced by mutation of San1, a nuclear RING finger ubiquitin ligase that has been implicated in the turnover of ts proteins in the nucleus [16] (Figure 1C, D). Though Ura3 is primarily a cytoplasmic protein, it appears that there is sufficient contact between San1 and Ura3-GFP to allow ubiquitination and subsequent proteasomal degradation of the fusion protein. The enhanced dependence on San1 shown by the GFP fusions may relate to a weak affinity of GFP for the nucleus. Indeed, microscopic examination showed that Ura3-GFP was present throughout the cytoplasm and nucleus, though it was excluded from vacuoles (data not shown; see also below).

Doa10 is an ER membrane protein, and may share some functions with Hrd1, the other major ER ubiquitin ligase. However, Ura3-3(GFP) was efficiently degraded in a *hrd1* deletion mutant, and as efficiently in a *hrd1 doa10* double mutant as in the *doa10* single mutant (Figure 1D). It appears therefore that this protein is not a substrate for Hrd1.

### Fusion of temperature-sensitive proteins to SNAREs

**Figure 2:**
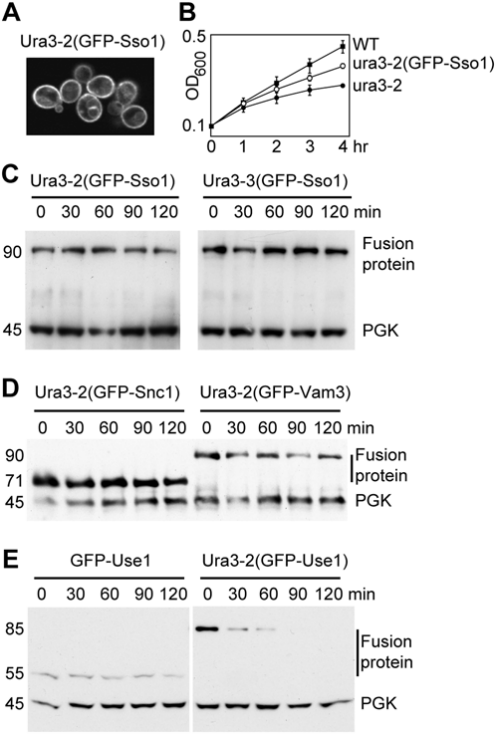
Fusion of Ura3 mutants to membrane proteins. A. Fluorescence image of cells expressing Ura3-2 fused to GFP-Sso1, after incubation for two hours at 37°C. B. Growth curves of wild-type cells and those expressing soluble Ura3-2 or the same protein fused to GFP-Sso1. Mean and standard deviation of triplicate samples are shown. Note optical density is log scale. Cells were shifted to 37°C at time zero but there is a lag before growth slows, presumably due to pools of uracil. The membrane tethered form remained ts, though with a slightly longer lag. Similar results were obtained with Ura3-3. C. Cycloheximide chase of the Sso1 fusions at 37°C. D. Cycloheximide chase of equivalent fusions of Ura3-2 to Snc1 and Vam3. E. Cycloheximide chase of GFP-Use1 with and without Ura3-2 attached to it. Numbers to the left of the blots indicate the actual molecular weights of the proteins (kDa). SNARE fusions typically migrate more slowly than standard molecular weight markers, appearing about 10 kDa larger than they are.

Having established that the ts alleles of Ura3 could induce degradation of attached proteins, we fused the mutant Ura3-GFP chimeras to the plasma membrane SNARE protein Sso1. Fluorescence microscopy confirmed that the bulk of the Ura3-2(GFP-Sso1) construct was indeed at the plasma membrane (Figure 2A). Cells expressing these constructs remained temperature-sensitive for growth in the absence of uracil (Figure 2B). Strikingly, however, temperature shift did not induce degradation of the resultant fusion proteins (Figure 2C).

We extended these studies by fusing Ura3-2(GFP) to three more SNARES: Snc1, also found mainly on the plasma membrane; Vam3, found on the vacuolar membrane; and Use1, an ER resident SNARE. The Snc1 and Vam3 fusions also avoided degradation after temperature shift (Figure 2D). In contrast, although GFP-Use1 itself was stable, addition of the Ura3-2 protein resulted in rapid degradation at 37°C (Figure 2E). Thus, a SNARE fusion can be degraded, but apparently only if it is in the ER where quality control of membrane proteins typically occurs. When located on the plasma membrane the fusion proteins escape proteasome-mediated degradation, even though they are exposed to the cytoplasm. Furthermore, though some protein could be seen on the vacuolar membrane, even after prolonged incubation at 37° there was no sign of GFP accumulation within the vacuole (Figure 2A). Nor could we detect free GFP by immunoblotting (not shown); this is a characteristic product of the vacuolar degradation of fusion proteins since GFP itself is protease resistant.

### Membrane attachment affects degradation of a ts lambda repressor

**Figure 3:**
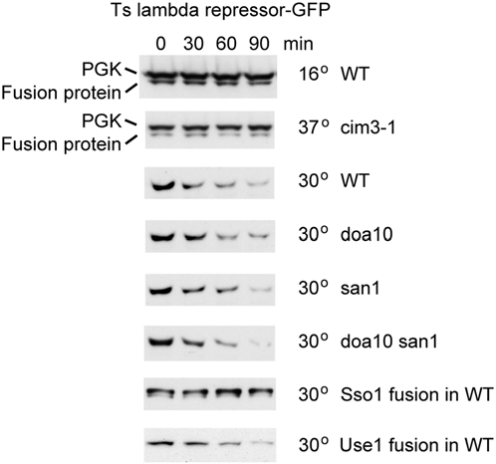
Degradation of a temperature-sensitive lambda repressor allele is affected by membrane attachment. The mutant repressor was fused to soluble GFP or to GFP-Sso1 (Sso1 fusion) or GFP-Use1 (Use1 fusion) and cyclohemimide chases performed with the indicated strains and temperatures. The PGK control was only probed in the top two panels, due to its poor separation from the fusion protein.

As an independent test of this phenomenon, we used a mutant (L57A, A66T) of the N-terminal 92 amino-acid region of the bacteriophage lambda repressor. The A66T mutation is present in the c1857 temperature-sensitive repressor [22], and the L57A mutation has been shown to induce physical unfolding at temperatures above 20°C [23]. Unlike the Ura3 mutants, this protein is monomeric and it should also unfold at normal yeast growth temperatures, thus avoiding any potential complications due to heat stress of the cells. A GFP fusion to the mutant repressor was stable at 16°, but was degraded at 30° in wild type cells. It was not degraded in the *cim3-1* proteasome mutant, but was degraded in cells lacking Doa10, San1 or both (Figure 3). Thus, it is likely to be a substrate for one or more ubiquitin ligases that do not recognise the Ura3 mutants, or possibly for the proteasome itself.

Despite these differences between the properties of the lambda repressor and Ura3 mutants, fusion of the repressor-GFP construct to Sso1 inhibited its degradation, just as with the Ura3 mutants (Figure 3). However, equivalent fusions to the ER SNARE Use1 were degraded, indicating that they were capable of becoming substrates for the ER-associated degradation machinery (Figure 3).

### Localisation of misfolded proteins

It has been reported that some abnormal proteins, such as a temperature sensitive mutant of Ubc9, accumulate in discrete punctae prior to degradation [19]. If this is a necessary step, it could explain why membrane-tethered proteins, which presumably are restricted in their movement, suffer a different fate from soluble ones. We therefore investigated the distribution of our soluble GFP chimeras following temperature shift.

**Figure 4:**
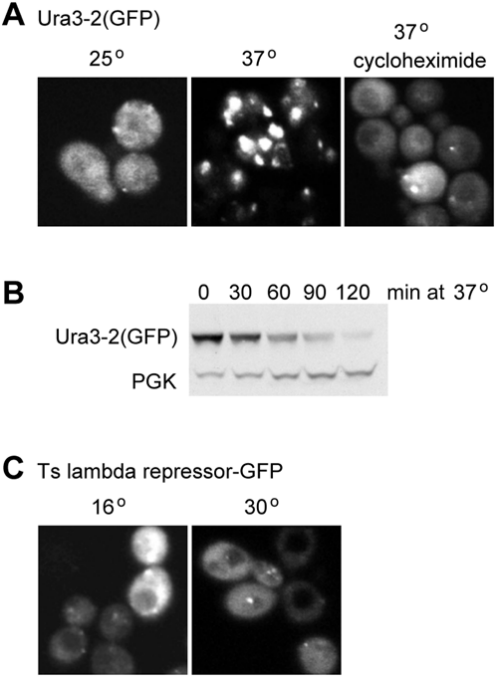
Aggregation does not correlate with degradation. A. GFP-tagged Ura3-2 was imaged in cells incubated at 25°C or 30 mins after a shift to 37°C, in the presence or absence of cycloheximide. B. GFP-tagged Ura3-2 was expressed from the *GAL* promoter, then expression repressed by growth in glucose prior to temperature shift, in the absence of cycloheximide. Degradation was normal even though the aggregation state of the protein was different from that in cycloheximide. C. GFP-tagged lambda repressor in cells gown at 16°C, or one hour after shifting to 30°C. The images have been approximately normalised for intensity.

The Ura3-2(GFP) construct was quite uniformly distributed at 25°, with only an occasional bright spot (Figure 4A). However, within 30 minutes of shifting the temperature to 37° in at least 80–90% of the cells the majority of the protein was in large fluorescent spots, consistent with aggregation. Surprisingly, however, this aggregation was almost completely prevented by the addition of cycloheximide prior to the temperature shift (Figure 4A). This dramatic difference led us to investigate whether the degradation of the protein was affected by cycloheximide, which is used in our standard assay. We used the glucose-repressible *GAL1* promoter to drive synthesis of Ura3-2(GFP) at low temperature, added glucose to repress transcription for four hours, then subjected the cells to high temperature. Figure 4B shows that the fusion protein was degraded just as it was in the presence of cycloheximide.

We also examined the GFP-tagged lambda repressor mutant, and found that it had a dispersed distribution with a few small bright dots, whether the cells were kept at 16° or had been shifted to 30° for one hour (Figure 4C). Taken together, these results indicate that the distribution of our mutant proteins does not correlate with their metabolic stability. There is thus little reason to believe that substantial aggregation or specific localisation is an obligatory or regulating step in their destruction. We cannot, of course, rule out the possibility that small or transient clusters are a necessary intermediate.

## Discussion

Our goal was to test how yeast cells deal with membrane proteins whose cytoplasmic domain becomes damaged. Using two temperature-sensitive mutants of Ura3 and one of the lambda repressor, all of which are degraded at high temperature when in soluble form, we reached the surprising conclusion that plasma or vacuolar membrane proteins bearing misfolded domains are not rapidly degraded either by the proteasome system or the vacuolar system. However, when attached to the ER, the domains do trigger rapid proteasomal destruction. It seems that cells rely on the ERAD system to catch misfolded proteins before they escape to other organelles, and have no special way to remove them if they misfold after reaching their destination.

It seems likely that the most common problem with membrane proteins is a failure to fold initially, in which case they are subject to ERAD. It may be that misfolding at a later stage is uncommon, and since the proteins we have studied do not aggregate when membrane-tethered, they may not be particularly harmful to the cell. Perhaps, therefore, there is no need for machinery to remove them.

What is curious, however, is that the proteins are not degraded even though they are exposed to the cytosol, and when free to move in the cytosol are good substrates for degradation. We considered several possible explanations for this. One is that some form of aggregation or localisation is necessary for degradation, but is prevented by membrane tethering. However, we could find little evidence that degradation of the soluble forms of the proteins was linked to their localisation. Second, the membrane proteins may be inaccessible to the ubiquitin ligases that recognise unfolded proteins. This is a plausible explanation, since the Ura3 mutants required either Doa10, an ER membrane protein, or San1, a nuclear protein, for their degradation. These ligases do seem to be major contributors to quality control, since several nuclear temperature-sensitive proteins are San1 substrates [16], and in a previous study peptide fusions to Ura3 that were selected for their instability all turned out to be Doa10 substrates [21]. Nevertheless, the lambda repressor mutant was degraded even in the absence of Doa10 and San1, yet was still rescued by membrane tethering. Thus, there must be at least one other enzyme involved in the turnover of unfolded proteins, and it too is evidently unable to mediate the degradation of plasma membrane proteins.

A third possibility is that the location next to a membrane is a protected environment, from which ubiquitin ligases and/or proteasomes are excluded, except for the ER where specific targeting mechanisms overcome this. It is notable that specific machinery is thought to recruit proteasomes to the ER membrane as well as extract membrane proteins [6], and such components may be absent from the plasma membrane. Interestingly, however, at least one protein of the outer mitochondrial membrane has been reported to be degraded by the ubiquitin-proteasome system [24], so the ER may not be unique in this respect.

It is also possible that quality control at the plasma membrane is actively countered, for example by localised chaperones or deubiquitinating enzymes. Deubiquitination has in fact been proposed to explain why the Pma1-7 mutant of the plasma membrane ATPase, which is normally ubiquitinated and routed from Golgi to vacuole, appears stable if allowed to reach the plasma membrane [25]. Efficient re-folding seems unlikely, given that Ura3 activity remains temperature sensitive when tethered to the membrane. We have subsequently directly screened mutagenised versions of tethered Ura3 and obtained additional alleles that were very tightly temperature-restricted for growth without uracil, but again none of these were degraded at high temperature.

It may be that the unfolded proteins are ubiquitinated on the plasma membrane, but escape degradation simply because they cannot be extracted from this membrane and are poor substrates for endocytosis. It could be, for example, that K48-linked ubiquitin chains are not well recognised by the endocytic machinery and instead are removed by deubiquitinating enzymes. If so, one might expect ubiquinated forms of the ts proteins to appear after temperature shift. We did in fact detect ubiquitinated forms of the ts Ura3(GFP-Sso1) constructs. However, the appearance of these forms was not reproducibly temperature-dependent, and they represented a very minor fraction of the protein. We were therefore unable to rule out the alternative possibility that they comprised a minor fraction of constitutively misfolded, and thus non-fluorescent, protein that remains in the ER and undergoes ERAD. The question of ubiquitination at the plasma membrane is thus still open.

It remains to be seen whether the yeast results can be generalised to other species, but there are reasons to be cautious. For example, misfolded proteins are usually recognised by chaperone proteins, and in animal cells persistently misfolded proteins are thought to become substrates for a soluble chaperone-associated ubiquitin ligase termed CHIP [26]. However, yeast lacks a CHIP homologue, and thus such a mechanism may not apply.

It is possible, of course, that machinery exists in yeast to remove plasma membrane proteins with cytoplasmic domains that are more extensively damaged than is the case for the three ts proteins we have used, for example if they undergo extensive aggregation. Indeed, the temperature sensitive Pma1-10 protein has been reported to be degraded after arrival at the cell surface [27]. However, this protein may undergo misfolding of its transmembrane portion, which would allow it to be recognised by quality control components that monitor structure within the lipid bilayer. In this work we have focussed exclusively on independently-folded cytoplasmic domains. Our conclusion is that quality control for such domains either does not exist, or is much less stringent and effective than that for soluble proteins. One implication is that membrane tethering is a potential strategy for preventing degradation of unstable proteins.

## Materials and Methods

### Yeast strains

Yeast strains unless otherwise stated were from the Open Biosystems knockout collection in a BY4742 (*MATα uraΔ0 leu2Δ0 his3Δ1 lys2Δ0*) background. The *doa10 san1* double mutant was made by homologous recombination with a replacement of the *DOA10* open reading frame in the BY4742 *san1* knockout strain by a PCR product encoding the *Schizosaccharomyces pombe HIS5* gene. The *doa10 hrd1* double mutant was made similarly. The *cim3-1* and corresponding wild type strain [28] were obtained from Carl Mann (SBIGeM CEA/Saclay France).

### Plasmids

PmUra414 was made by insertion of a c-myc tagged PCR product from the *URA3* gene into a version of pRS414 bearing the *TPI1* promoter. The *ura3* mutants were made by error-prone PCR mutagenesis [29]. The mutated PCR products were co-transformed into SEY6210 (*MATα ura3-52 leu2-3, -112 his3-Δ200 trp1-Δ901 lys2-801 suc2-Δ9*) together with Nco1-Bpu101 cut pmUra414 and selected on plates lacking tryptophan. A pool of colonies was then plated onto 5-fluoroorotic acid plates at either 25°C or 37°C. Colonies from these plates were streaked out and further tested for temperature sensitive growth on plates lacking uracil. Rescued plasmids were sequenced.

In-frame GFP fusions were made between downstream EcoR1 and BamH1 sites. The SSO1 fusion was made by replacement of the GFP with a GFP-SSO1 fusion [30]. Fusions were similarly made to the N-termini of *SNC1* and *VAM3*. *USE1* was cloned by PCR into a *TPI1*-promoter-GFP containing version of pRS416 and the GFP-*USE1* fusion used to replace GFP in the *URA3* fusion constructs.

The region encoding the first 92 amino acids from the c1857 bacteriophage lambda repressor was cloned by PCR and subsequently mutated by PCR to introduce a L57A change [23]. This fragment was subsequently used to replace the *URA3* moiety in the GFP fusion constructs. Galactose-inducible constructs were made by placing the fusion constructs in a version of YCpLac33 carrying the *GAL1/10* promoter fragment. Sequences of all PCR generated constructs were verified by DNA sequencing.

### Cycloheximide chase experiments

Cells were grown into early log phase and after addition of cycloheximide (to 500 µg/ml) were shaken at the temperatures shown, and samples taken at the indicated times. These were spun down and stored on dry ice before being subjected to alkaline lysis and solublisation as described by [31]. Samples were run on PAGE and western blots probed with anti-GFP (Roche Diagnostics) and anti PGK (Molecular Probes). In experiments using the *cim3-1* strain, cells were preincubated for 1 hour at 37°C prior to the addition of cycloheximide. For the galactose shutoff experiments cells were transferred from medium containing 2% galactose and raffinose into medium containing 2% glucose for 4 hours before temperature shift.

### Imaging

Live cell imaging was performed on concanavalin-A coated slides using a Zeiss LSM510. Images were adjusted for contrast and brightness, and in some cases, they were blurred to filter noise, by using Adobe Photoshop (Adobe Systems, Mountain View, CA).
